# Supplementary material for: Combining the Δ-Self-Consistent-Field and GW Methods for Predicting Core Electron Binding Energies in Periodic Solids
Source: J Chem Theory Comput. 2023 May 10;19(11):3276–83. doi: 10.1021/acs.jctc.3c00121 (PMC10269324; doi:10.1021/acs.jctc.3c00121)

Supplementary Information for

Combining the Δ-Self-Consistent-Field and GW Methods for Predicting Core Electron Binding Energies in Periodic Solids

J. Matthias Kahk[†] and Johannes Lischner[‡]

[†]Institute of Physics, University of Tartu, W. Ostwaldi 1, 50411 Tartu, Estonia

email: juhan.matthias.kahk@ut.ee

[‡]Department of Physics and Department of Materials, and the Thomas Young Centre for Theory and Simulation of Materials, Imperial College London, London SW7 2AZ, United Kingdom

Table of Contents

Binding energies from Eqn. 1 and Eqn. 3, extrapolation....3
BeO....3
Beryllium....4
β-SiC....5
Diamond....6
Graphite....7
hex-BN....8
Lithium....9
Magnesium....10
Silicon....11
Sodium....12
Numerical verification of Eqn. 2....13
Structures and k-point grids used in the GW and GWΓ calculations....15
BeO....15
Be metal....15
β-SiC....15
Diamond....15
Graphite....16
hex-BN....16
Li metal....16
Mg metal....16
Silicon....17
Na metal....17
Extrapolation of the GW and GWΓ results to $E_{cut} \Rightarrow +\infty$....18
BeO....18
Be metal....18
β-SiC....19
Diamond....19
Graphite....20
hex-BN....20
Li metal....21
Mg metal....21
Silicon....22
Na metal....22

Binding energies from Eqn. 1 and Eqn. 3, extrapolation

BeO

Supercell	Atoms per supercell	(Atoms per supercell)^(-1/3)	O 1s B.E., Eqn. 1, PBE	O 1s B.E., Eqn. 3, PBE	Be 1s B.E., Eqn. 1, PBE	Be 1s B.E., Eqn. 3, PBE
1x1x1	4	0.630	525.75	526.66	107.82	108.74
2x2x1	16	0.397	526.56	526.91	108.80	109.14
3x3x2	72	0.240	527.24	527.39	109.48	109.62
4x4x3	196	0.172	527.52	527.62	109.76	109.86
5x5x3	300	0.149	527.60	527.68	109.84	109.93
6x6x3	432	0.132	527.67	527.75	109.92	109.99

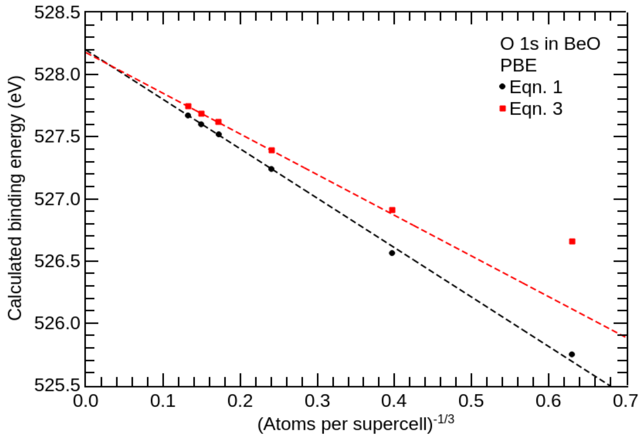

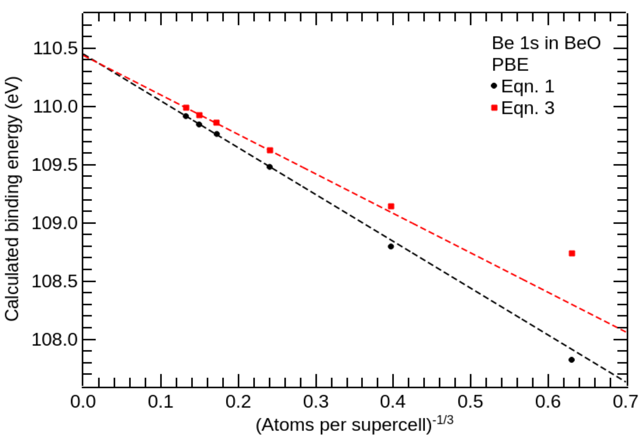

Supercell	Atoms per supercell	(Atoms per supercell)^(-1/3)	O 1s B.E., Eqn. 1, SCAN	O 1s B.E., Eqn. 3, SCAN	Be 1s B.E., Eqn. 1, SCAN	Be 1s B.E., Eqn. 3, SCAN
1x1x1	4	0.630	526.34	527.26	108.07	108.98
2x2x1	16	0.397	527.16	527.50	109.06	109.41
3x3x2	72	0.240	527.85	528.01	109.77	109.91
4x4x3	196	0.172	528.15	528.25	110.07	110.17
5x5x3	300	0.149	528.23	528.32	110.16	110.24
6x6x3	432	0.132	528.31	528.38	110.23	110.31

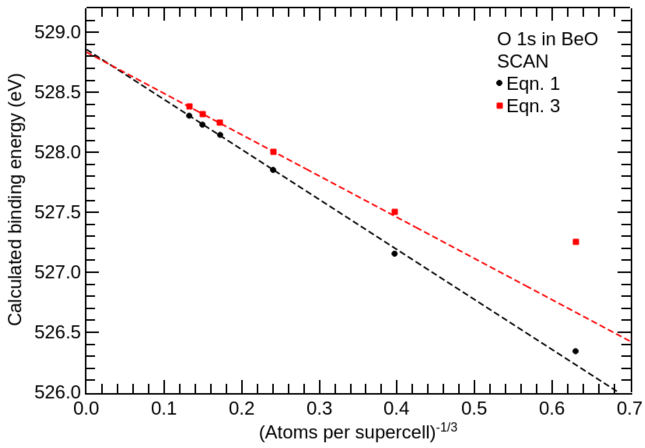

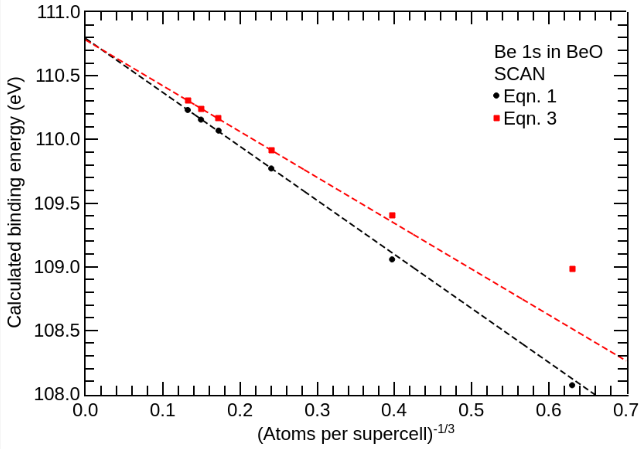

Beryllium

Supercell	Atoms per supercell	(Atoms per supercell)^(-1/3)	Be 1s B.E., Eqn. 1, PBE	Be 1s B.E., Eqn. 3, PBE	Be 1s B.E., Eqn. 1, SCAN	Be 1s B.E., Eqn. 3, SCAN
1x1x1	2	0.794	109.89	111.24	110.24	112.04
2x2x1	8	0.500	110.70	111.29	111.07	111.69
3x3x2	36	0.303	111.24	111.38	111.66	111.81
4x4x3	96	0.218	111.37	111.43	111.80	111.87
5x5x3	150	0.188	111.44	111.51	111.89	111.95
6x6x4	288	0.151	111.43	111.45	111.87	111.91

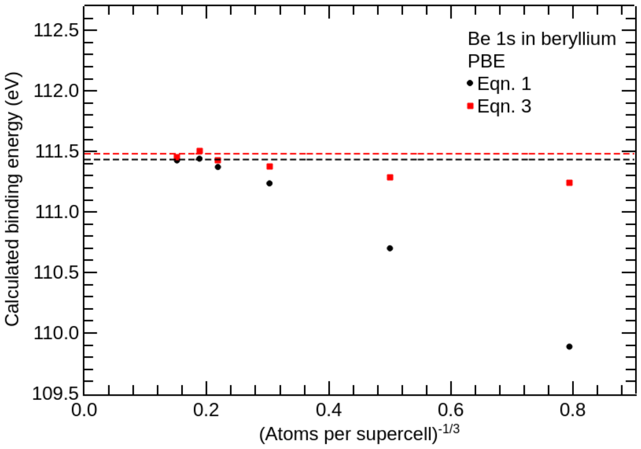

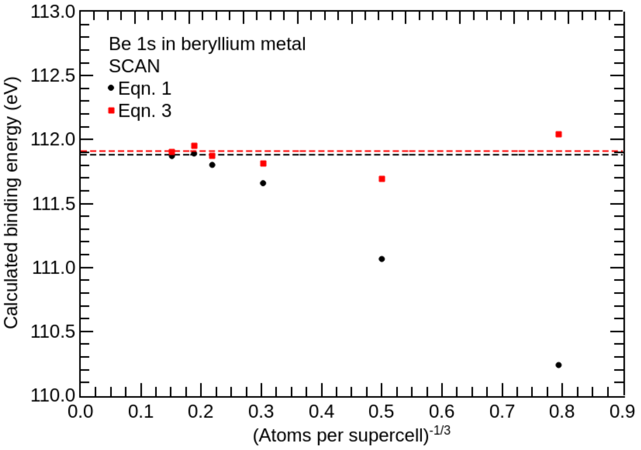

β-SiC

Supercell	Atoms per supercell	(Atoms per supercell)^(-1/3)	C 1s B.E., Eqn. 1, PBE	C 1s B.E., Eqn. 3, PBE	Si 2p B.E., Eqn. 1, PBE	Si 2p B.E., Eqn. 3, PBE
1x1x1	2	0.794	279.22	280.40	96.82	98.00
2x2x2	16	0.397	280.32	280.64	98.22	98.53
3x3x3	54	0.265	280.52	280.55	98.34	98.48
4x4x4	128	0.198	280.62	280.72	98.44	98.54
5x5x5	250	0.159	280.68	280.76	98.51	98.58

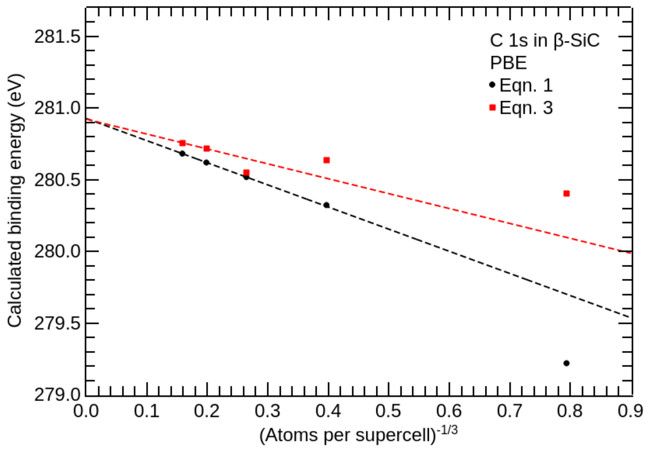

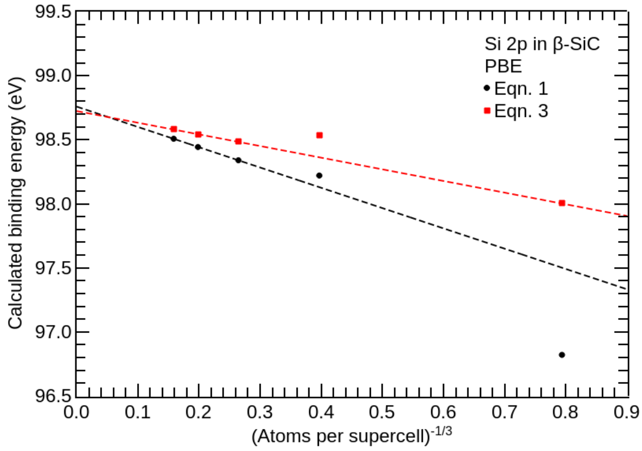

Supercell	Atoms per supercell	(Atoms per supercell)^(-1/3)	C 1s B.E., Eqn. 1, SCAN	C 1s B.E., Eqn. 3, SCAN	Si 2p B.E., Eqn. 1, SCAN	Si 2p B.E., Eqn. 3, SCAN
1x1x1	2	0.794	279.68	280.88	98.42	99.62
2x2x2	16	0.397	280.82	281.14	98.50	98.82
3x3x3	54	0.265	281.04	281.19	98.76	98.91
4x4x4	128	0.198	281.15	281.25	98.88	98.97
5x5x5	250	0.159	281.22	281.29	98.95	99.02

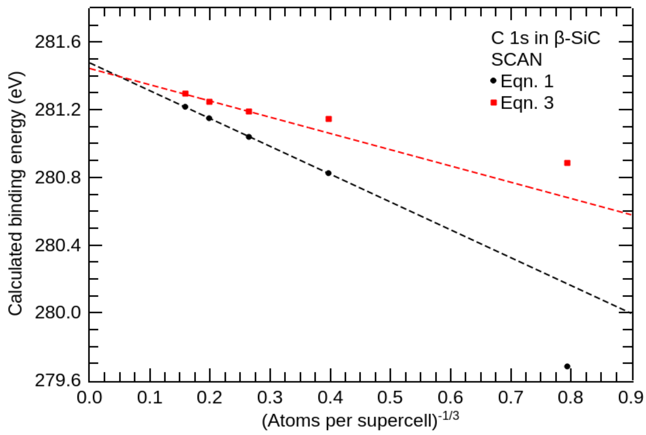

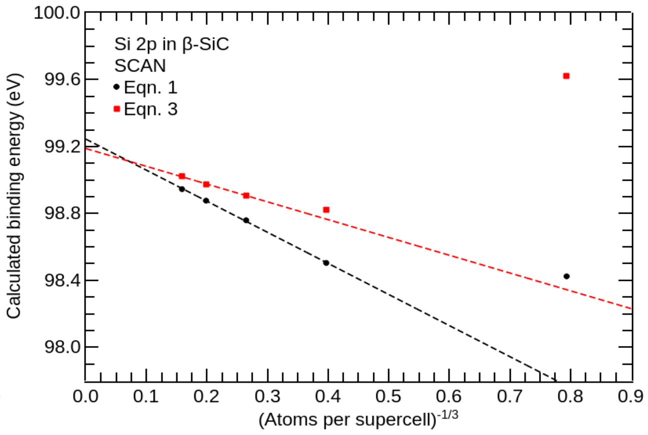

Diamond

Supercell	Atoms per supercell	(Atoms per supercell)^(-1/3)	C 1s B.E., Eqn. 1, PBE	C 1s B.E., Eqn. 3, PBE	C 1s B.E., Eqn. 1, SCAN	C 1s B.E., Eqn. 3, SCAN
1x1x1	2	0.794	280.34	282.86	280.93	283.48
2x2x2	16	0.397	282.55	283.33	283.11	283.91
3x3x3	54	0.265	283.07	283.44	283.66	283.99
4x4x4	128	0.198	283.31	283.52	283.81	284.09
5x5x5	250	0.159	283.45	283.59	284.00	284.15
6x6x6	432	0.132	283.51	283.62	284.05	284.17

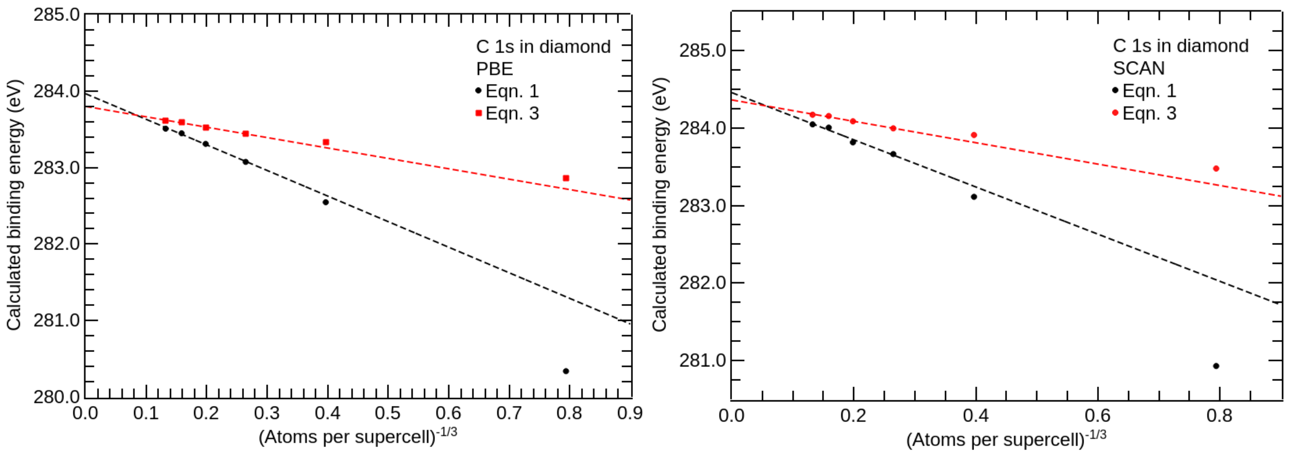

Graphite

Supercell	Atoms per supercell	(Atoms per supercell)^(-1/3)	C 1s B.E., Eqn. 1, PBE	C 1s B.E., Eqn. 3, PBE	C 1s B.E., Eqn. 1, SCAN	C 1s B.E., Eqn. 3, SCAN
1x1x1	4	0.630	282.39	284.26	283.26	285.11
2x2x2	32	0.315	283.10	283.78	283.92	284.61
3x3x3	108	0.210	283.28	283.62	284.09	284.44
4x4x4	256	0.157	283.37	283.56	284.18	284.38
2x2x1	16	0.397	282.48	283.45	283.24	284.22
4x4x2	128	0.198	283.14	283.45	283.95	284.26
6x6x3	432	0.132	283.30	283.45	284.11	284.26

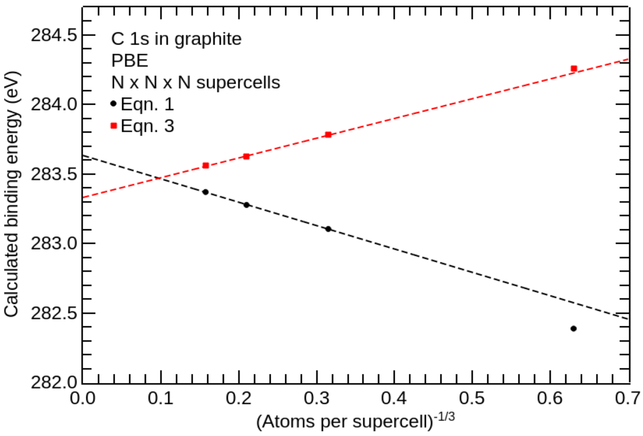

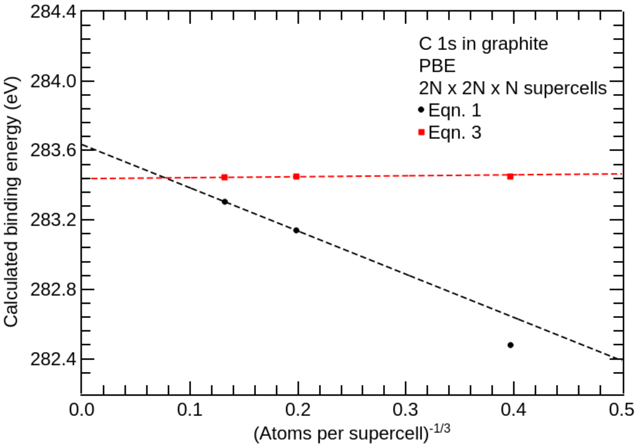

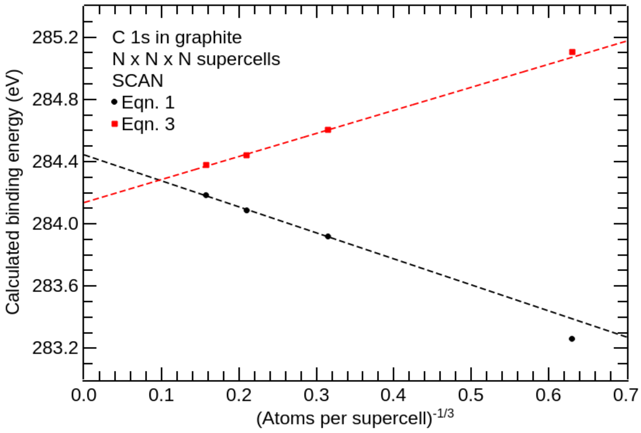

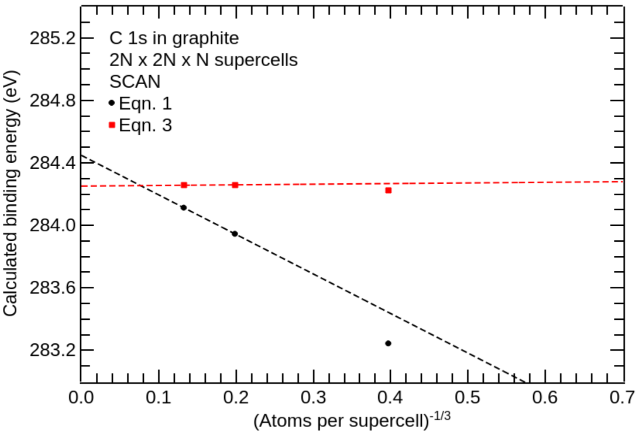

hex-BN

Supercell	Atoms per supercell	(Atoms per supercell)^(-1/3)	B 1s B.E., Eqn. 1, PBE	B 1s B.E., Eqn. 3, PBE	N 1s B.E., Eqn. 1, PBE	N 1s B.E., Eqn. 3, PBE
2x2x1	16	0.397	187.14	187.41	394.93	395.20
4x4x2	128	0.198	187.42	187.48	395.34	395.40
6x6x3	432	0.132	187.54	187.56	395.48	395.50

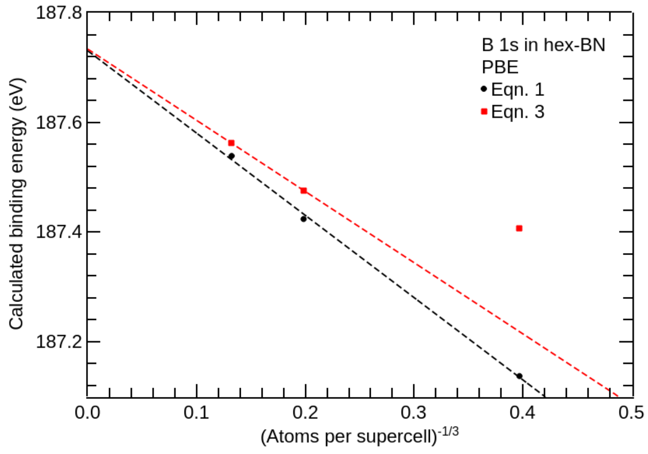

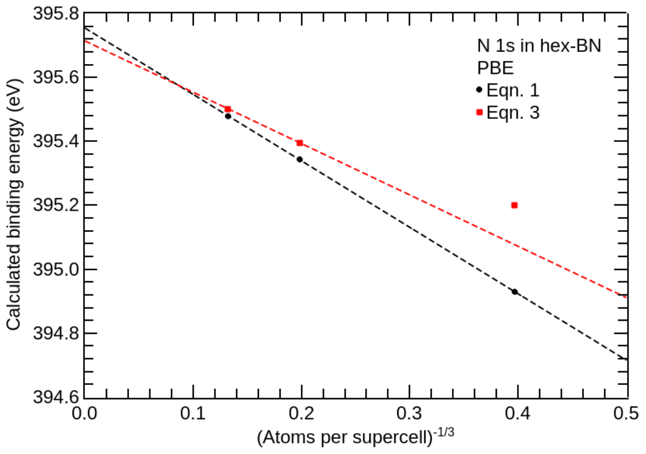

Supercell	Atoms per supercell	(Atoms per supercell)^(-1/3)	B 1s B.E., Eqn. 1, SCAN	B 1s B.E., Eqn. 3, SCAN	N 1s B.E., Eqn. 1, SCAN	N 1s B.E., Eqn. 3, SCAN
2x2x1	16	0.397	187.81	188.07	395.54	395.80
4x4x2	128	0.198	188.10	188.15	395.96	396.01
6x6x3	432	0.132	188.22	188.25	396.11	396.13

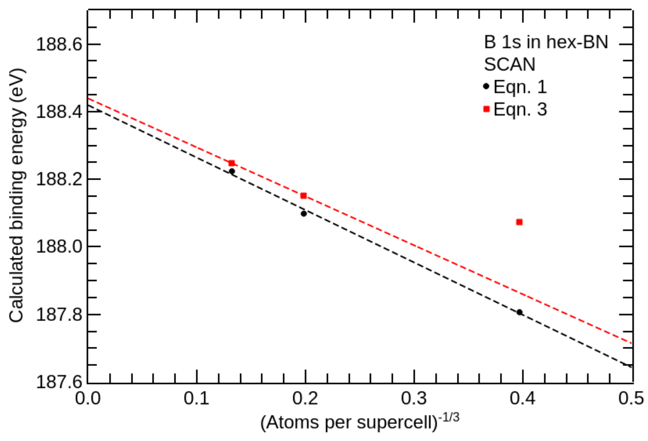

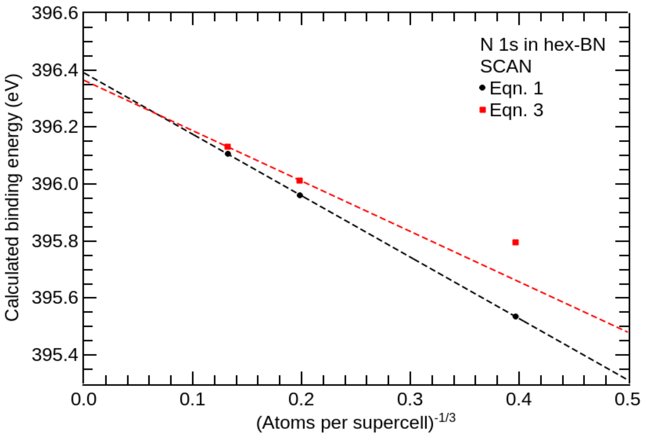

Lithium

Supercell	Atoms per supercell	(Atoms per supercell)^(-1/3)	Li 1s B.E., Eqn. 1, PBE	Li 1s B.E., Eqn. 3, PBE	Li 1s B.E., Eqn. 1, SCAN	Li 1s B.E., Eqn. 3, SCAN
1x1x1	1	1.000	54.73	54.83	55.08	55.15
2x2x2	8	0.500	54.64	54.65	54.57	54.57
3x3x3	27	0.333	54.65	54.64	54.88	54.88
4x4x4	64	0.250	54.65	54.64	54.88	54.88
5x5x5	125	0.200	54.64	54.64	54.87	54.86

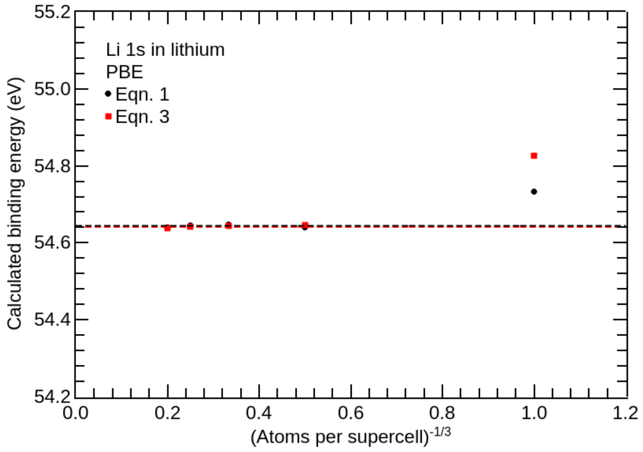

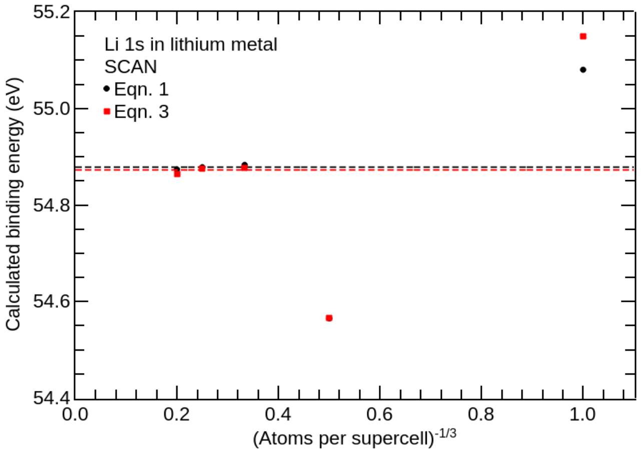

Magnesium

Supercell	Atoms per supercell	(Atoms per supercell)^(-1/3)	Mg 1s B.E., Eqn. 1, PBE	Mg 1s B.E., Eqn. 3, PBE	Mg 2p B.E., Eqn. 1, PBE	Mg 2p B.E., Eqn. 3, PBE
1x1x1	2	0.794	1300.48	1300.83	43.16	43.51
2x2x1	8	0.500	1300.75	1300.84	49.26	49.34
3x3x2	36	0.303	1300.84	1300.87	49.39	49.41
4x4x3	96	0.218	1300.87	1300.88	49.42	49.43
5x5x3	150	0.188	1300.87	1300.88	49.42	49.43
6x6x4	288	0.151	1300.87	1300.88	49.43	49.44

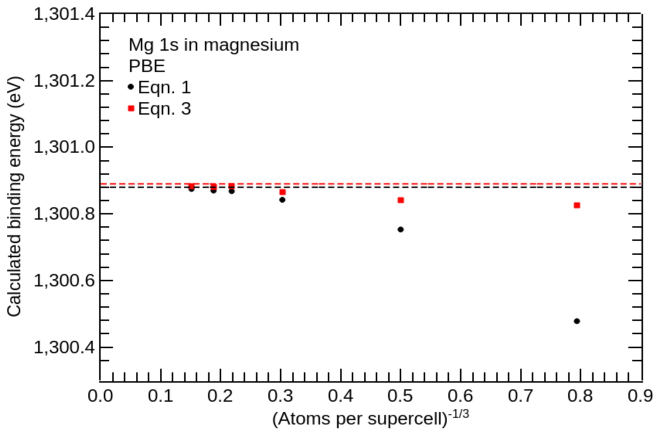

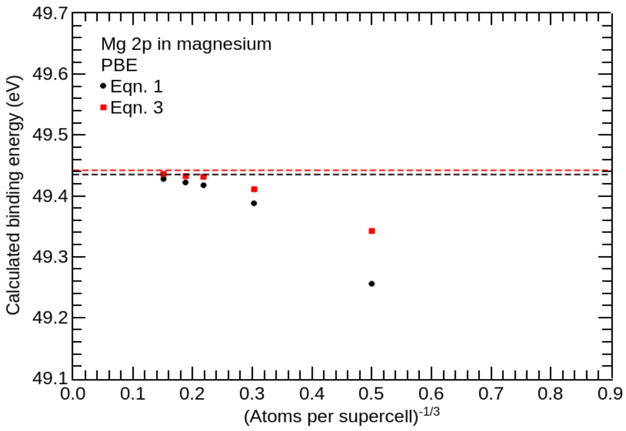

Supercell	Atoms per supercell	(Atoms per supercell)^(-1/3)	Mg 1s B.E., Eqn. 1, SCAN	Mg 1s B.E., Eqn. 3, SCAN	Mg 2p B.E., Eqn. 1, SCAN	Mg 2p B.E., Eqn. 3, SCAN
1x1x1	2	0.794	1302.85	1303.21	50.16	50.52
2x2x1	8	0.500	1303.11	1303.20	49.52	49.61
3x3x2	36	0.303	1303.22	1303.24	49.63	49.65
4x4x3	96	0.218	1303.24	1303.23	49.69	49.68
5x5x3	150	0.188	1303.25	1303.26	49.68	49.70
6x6x4	288	0.151	1303.25	1303.26	49.70	49.71

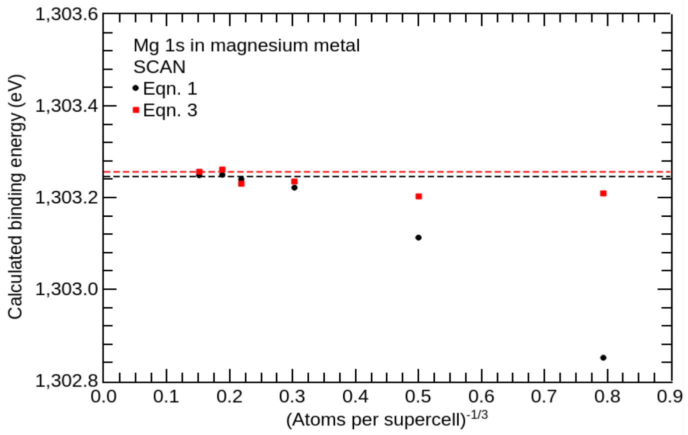

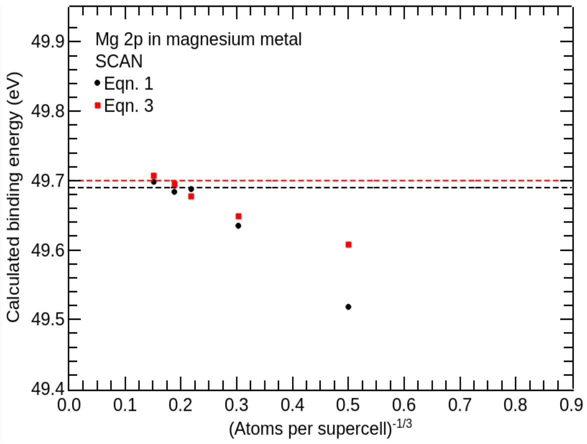

Silicon

Supercell	Atoms per supercell	(Atoms per supercell)^(-1/3)	Si 2p B.E., Eqn. 1, PBE	Si 2p B.E., Eqn. 3, PBE	Si 2p B.E., Eqn. 1, SCAN	Si 2p B.E., Eqn. 3, SCAN
1x1x1	2	0.794	97.11	98.22	97.45	98.57
2x2x2	16	0.397	98.26	98.64	98.63	99.00
3x3x3	54	0.265	98.43	98.61	98.80	98.98
4x4x4	128	0.198	98.51	98.61	98.89	98.99
5x5x5	250	0.159	98.55	98.62	98.96	99.04

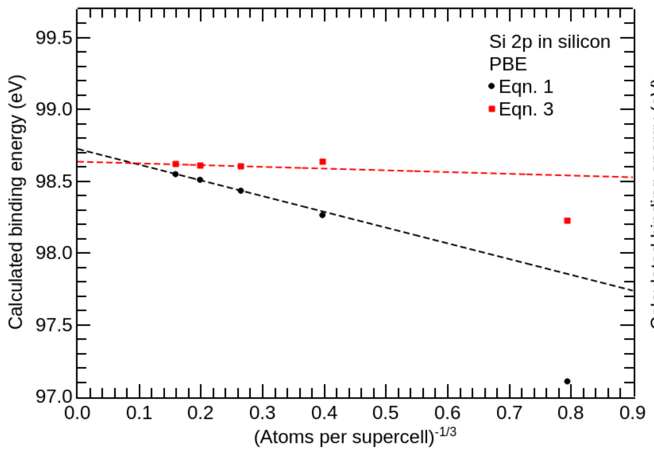

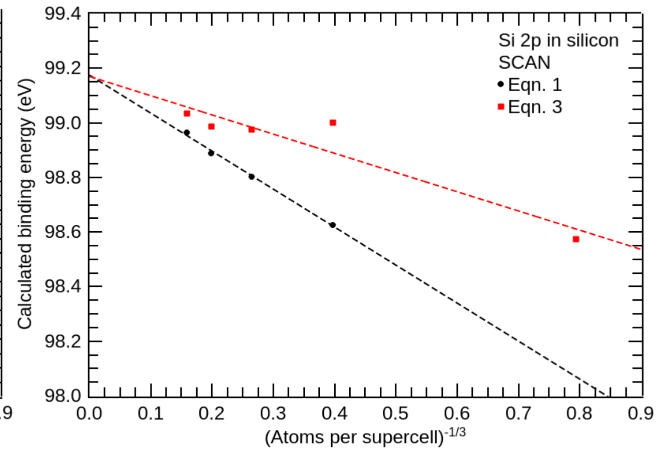

Sodium

Supercell	Atoms per supercell	(Atoms per supercell)^(-1/3)	Na 1s B.E., Eqn. 1, PBE	Na 1s B.E., Eqn. 3, PBE	Na 2p B.E., Eqn. 1, PBE	Na 2p B.E., Eqn. 3, PBE
1x1x1	1	1.000	1069.45	1069.61	30.51	30.67
2x2x2	8	0.500	1069.65	1069.67	30.54	30.56
3x3x3	27	0.333	1069.67	1069.68	30.57	30.58
4x4x4	64	0.250	1069.68	1069.68	30.59	30.59
5x5x5	125	0.200	1069.68	1069.68	30.58	30.59

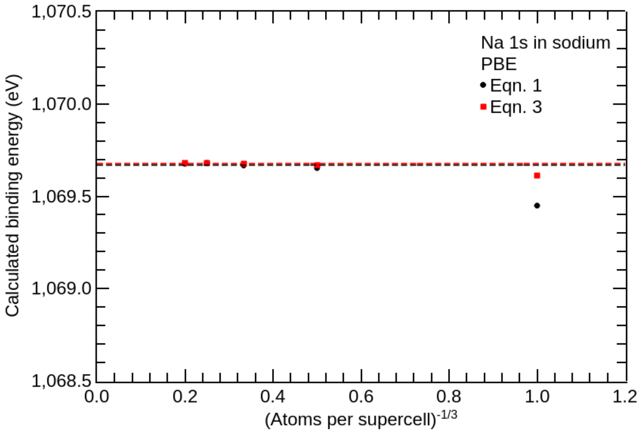

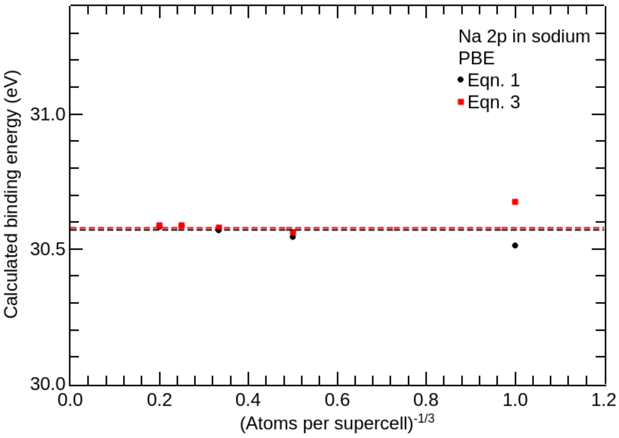

Supercell	Atoms per supercell	(Atoms per supercell)^(-1/3)	Na 1s B.E., Eqn. 1, SCAN	Na 1s B.E., Eqn. 3, SCAN	Na 2p B.E., Eqn. 1, SCAN	Na 2p B.E., Eqn. 3, SCAN
1x1x1	1	1.000	1071.37	1071.51	31.48	31.63
2x2x2	8	0.500	1071.55	1071.56	30.63	30.65
3x3x3	27	0.333	1071.56	1071.57	30.64	30.65
4x4x4	64	0.250	1071.58	1071.58	30.65	30.66
5x5x5	125	0.200	1071.57	1071.58	30.65	30.66

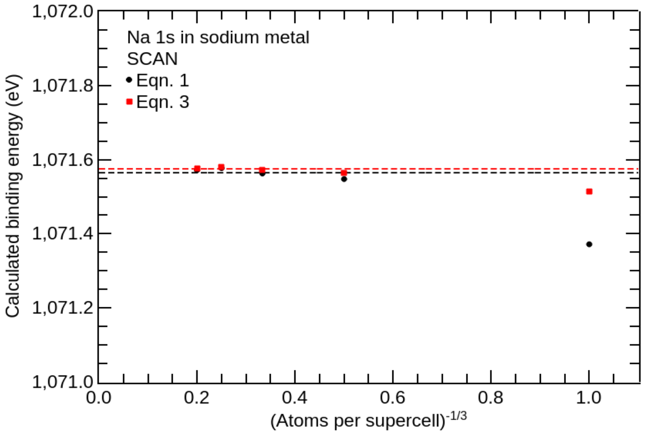

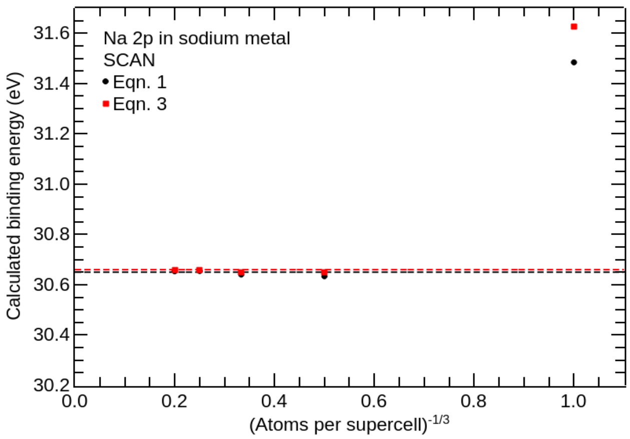

Numerical verification of Eqn. 2

Plots of the difference between the ΔSCF result for the first ionization energy, and the negative Kohn-Sham eigenvalue of the highest occupied state, as a function of the inverse cube root of the number of atoms per supercell, are provided for all materials considered in this work.

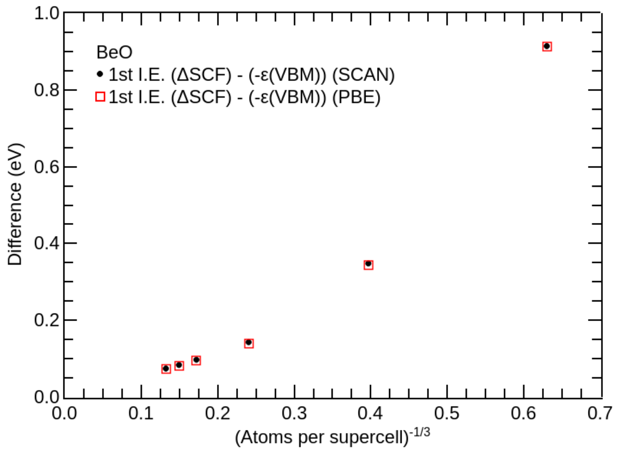

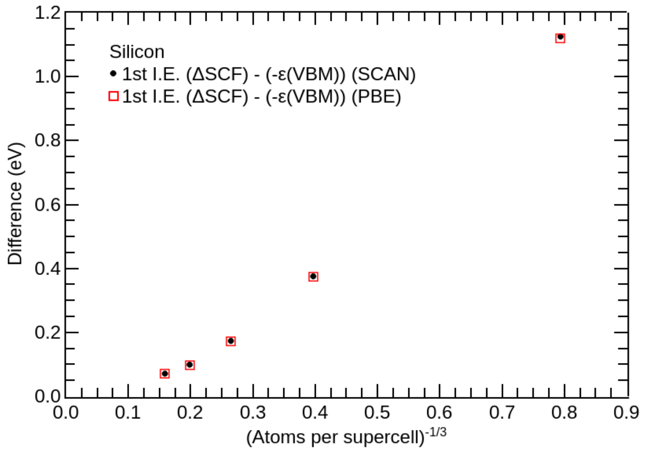

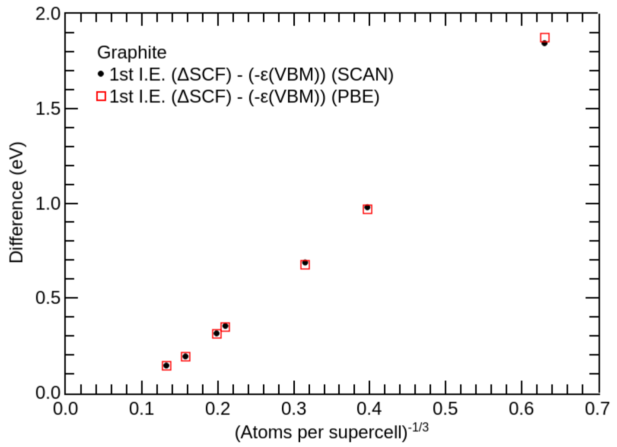

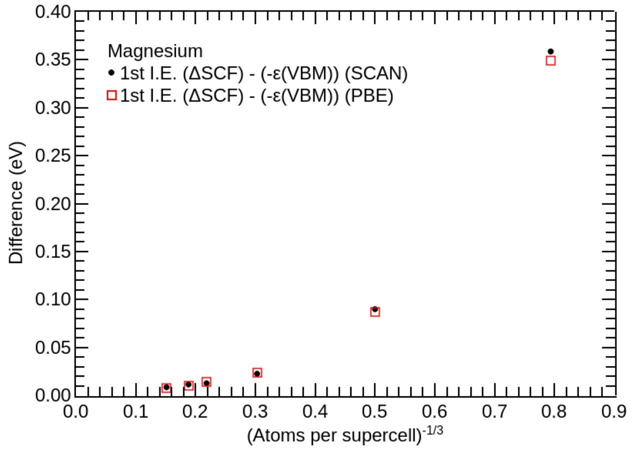

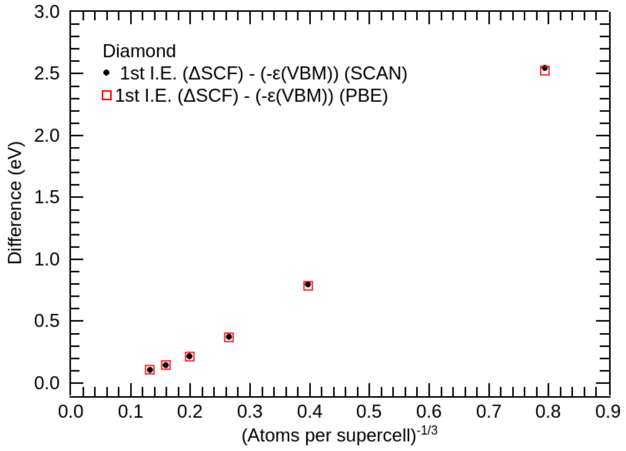

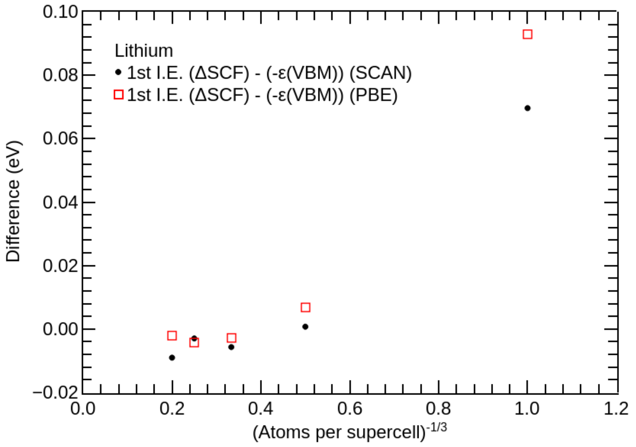

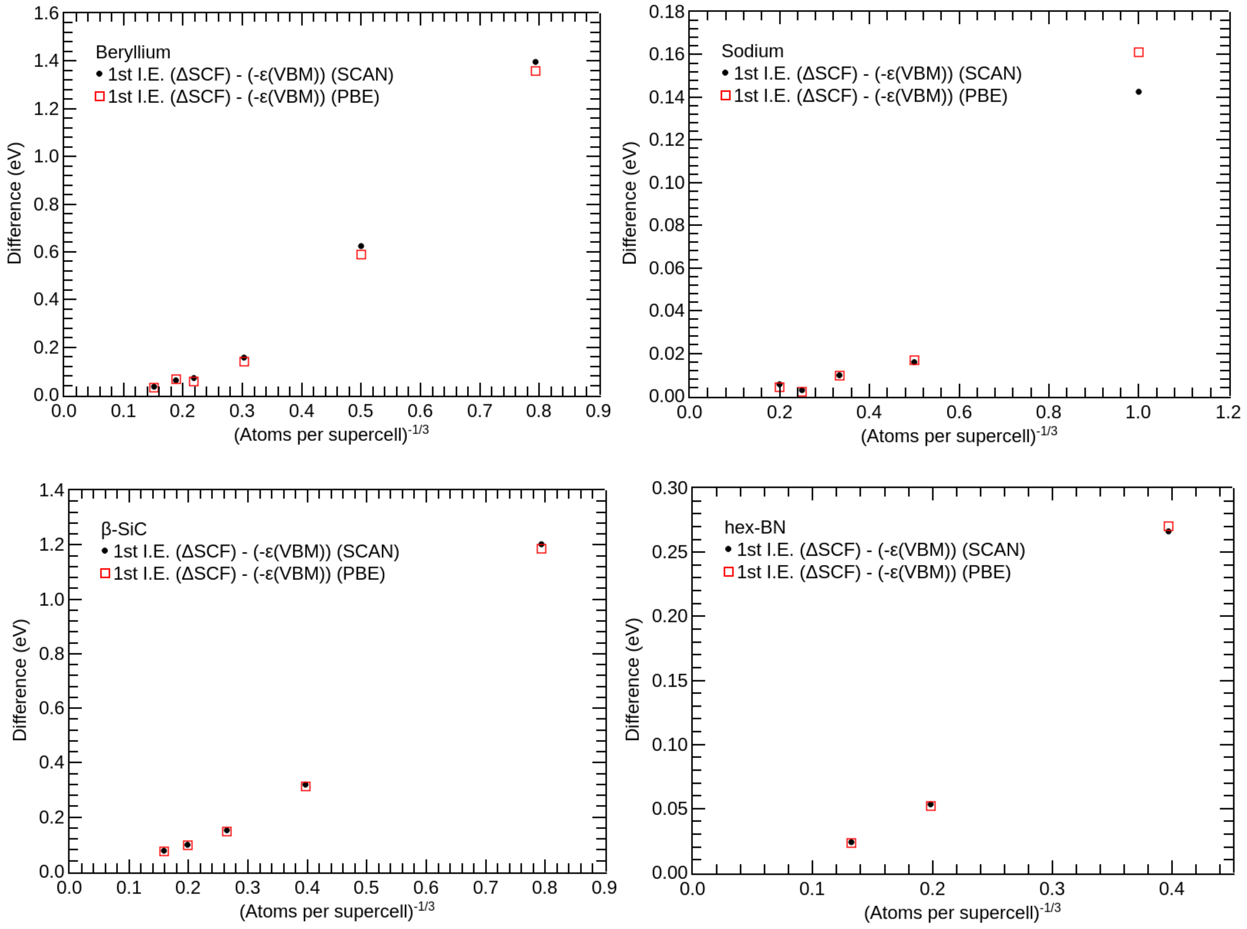
Beryllium
1st I.E. (ΔSCF) - (-ε(VBM)) (SCAN)
1st I.E. (ΔSCF) - (-ε(VBM)) (PBE)
Difference (eV)
(Atoms per supercell)^-1/3
Sodium
1st I.E. (ΔSCF) - (-ε(VBM)) (SCAN)
1st I.E. (ΔSCF) - (-ε(VBM)) (PBE)
β-SiC
1st I.E. (ΔSCF) - (-ε(VBM)) (SCAN)
1st I.E. (ΔSCF) - (-ε(VBM)) (PBE)
hex-BN
1st I.E. (ΔSCF) - (-ε(VBM)) (SCAN)
1st I.E. (ΔSCF) - (-ε(VBM)) (PBE)

Structures and k-point grids used in the GW and GWΓ calculations

The lattice vectors and the atomic positions are given in units of Ångström.

BeO

```
lattice_vector  1.342104220     2.324592698     0.000000000
lattice_vector  1.342104220     -2.324592698    0.000000000
lattice_vector  0.000000000     0.000000000     -4.362848360
atom    1.342104220     -0.774864233    0.000000000     Be
atom    1.342104220     0.774864233     -2.181424180    Be
atom    1.342104220     -0.774864233    -2.711073971    O
atom    1.342104220     0.774864233     -0.529649791    O
```

k-point grid: (12, 12, 8)

Be metal

```
lattice_vector  2.261367683     0.000000000     0.000000000
lattice_vector  -1.130683842    1.958401861     0.000000000
lattice_vector  0.000000000     0.000000000     3.571042160
atom    0.000000000     1.305601241     2.678281620     Be
atom    1.130683842     0.652800620     0.892760540     Be
```

k-point grid: (16, 16, 9)

β-SiC

```
lattice_vector  2.170890371     2.170890371     0.000000000
lattice_vector  0.000000000     2.170890371     2.170890371
lattice_vector  2.170890371     0.000000000     2.170890371
atom    0.000000000     0.000000000     0.000000000     Si
atom    1.085445186     1.085445186     1.085445186     C
```

k-point grid: (12, 12, 12)

Diamond

```
lattice_vector  2.50306525298690      0.000000000000000      0.0000000000000
lattice_vector  1.25153262649345      2.167718096416780      0.0000000000000
lattice_vector  1.25153262649345      0.722572698805593      2.0437442209028
atom    0.625766313246725     0.361286349402796     0.25546802761285      C
atom    4.380364192727070     2.529004445819570     1.78827619328995      C
```

k-point grid: (12, 12, 12)

Graphite

```
lattice_vector  1.225034982     2.121822829     0.000000000
lattice_vector  1.225034982     -2.121822829    0.000000000
lattice_vector  0.000000000     0.000000000     -6.909437660
atom    0.000000000     0.000000000     -5.182078245    C
atom    0.000000000     0.000000000     -1.727359415    C
atom    1.225034982     -0.707274276    -5.182078245    C
atom    1.225034982     0.707274276     -1.727359415    C
```

k-point grid: (14, 14, 4)

hex-BN

```
lattice_vector  2.494340000     0.000000000     0.000000000
lattice_vector  -1.247170000    2.160161806     0.000000000
lattice_vector  0.000000000     0.000000000     6.756746360
atom    0.000000000     0.000000000     3.378373180     B
atom    0.000000000     1.440107870     0.000000000     B
atom    0.000000000     0.000000000     0.000000000     N
atom    0.000000000     1.440107870     3.378373180     N
```

k-point grid: (14, 14, 5)

Li metal

```
lattice_vector  3.481664463     0.000000000     0.000000000
lattice_vector  0.000000000     3.481664463     0.000000000
lattice_vector  0.000000000     0.000000000     3.481664463
atom    0.000000000     0.000000000     0.000000000     Li
atom    1.740832232     1.740832232     1.740832232     Li
```

k-point grid: (14, 14, 14)

Mg metal

```
lattice_vector  3.160811260     0.000000000     0.000000000
lattice_vector  -1.580405630    2.737342847     0.000000000
lattice_vector  0.000000000     0.000000000     5.163388560
atom    0.000000000     1.824895232     1.290847140     Mg
atom    1.580405630     0.912447616     3.872541420     Mg
```

k-point grid: (14, 14, 8)

Silicon

```
lattice_vector  0.000000000     2.716933420     2.716933420
lattice_vector  2.716933420     0.000000000     2.716933420
lattice_vector  2.716933420     2.716933420     0.000000000
atom    0.000000000     0.000000000     0.000000000     Si
atom    1.358466710     1.358466710     1.358466710     Si
```

k-point grid: (12, 12, 12)

Na metal

```
lattice_vector  4.192608683     0.000000000     0.000000000
lattice_vector  0.000000000     4.192608683     0.000000000
lattice_vector  0.000000000     0.000000000     4.192608683
atom    0.000000000     0.000000000     0.000000000     Na
atom    2.096304342     2.096304342     2.096304342     Na
```

k-point grid: (12, 12, 12)

Extrapolation of the GW and GWΓ results to $E_{cut} \Rightarrow +\infty$

BeO

E_{cut} (eV)	$E_{cut}^{-3/2}$ ($eV^{-3/2}$)	nbands	$\epsilon_{max,GW\Gamma}$ (eV)	$\epsilon_{max,GW}$ (eV)
300	0.0001925	321	3.032	2.898
350	0.0001527	404	3.029	2.834
400	0.0001250	494	3.008	2.786

Highest occupied state at k-point 0: (0.000, 0.000, 0.000)

$\epsilon_{max,PBE}$: 4.85 eV

Extrapolated $\epsilon_{max,GW\Gamma@PBE}$: 2.97 eV

Extrapolated $\epsilon_{max,GW@PBE}$: 2.58 eV

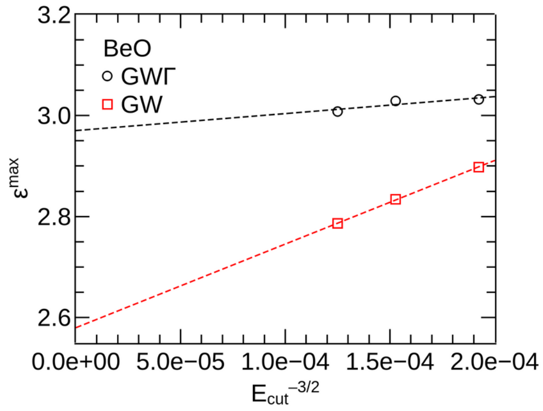

Be metal

E_{cut} (eV)	$E_{cut}^{-3/2}$ ($eV^{-3/2}$)	nbands	$\epsilon_{max,GW\Gamma}$ (eV)	$\epsilon_{max,GW}$ (eV)
300	0.0001925	186	7.93	7.233
350	0.0001527	235	7.936	7.226
400	0.0001250	287	7.929	7.222

Highest occupied state at k-point 151: (0.438, -0.125, 0.000)

$\epsilon_{max,PBE}$: 7.47 eV

Extrapolated $\epsilon_{max,GW\Gamma@PBE}$: 7.93 eV

Extrapolated $\epsilon_{max,GW@PBE}$: 7.20 eV

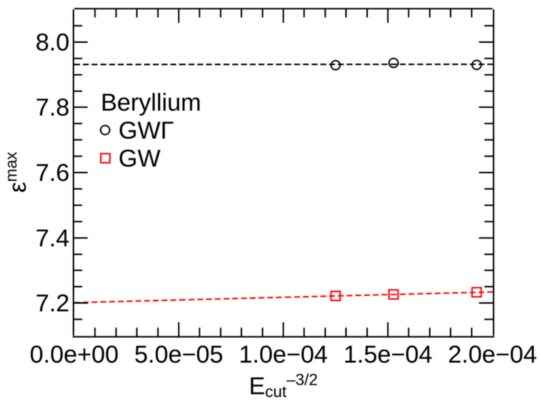

β-SiC

E_{cut} (eV)	$E_{cut}^{-3/2}$ ($eV^{-3/2}$)	nbands	$\epsilon_{max,GW\Gamma}$ (eV)	$\epsilon_{max,GW}$ (eV)
300	0.0001925	241	8.068	7.468
350	0.0001527	304	8.076	7.44
400	0.0001250	371	8.061	7.422

Highest occupied state at k-point 0: (0.000, 0.000, 0.000)

$\epsilon_{max,PBE}$: 8.38 eV

Extrapolated $\epsilon_{max,GW\Gamma@PBE}$: 8.06 eV

Extrapolated $\epsilon_{max,GW@PBE}$: 7.34 eV

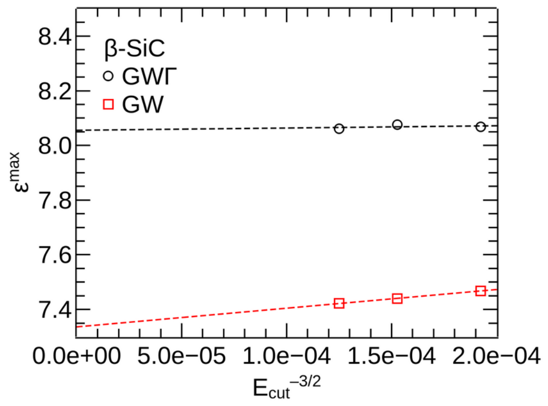

Diamond

E_{cut} (eV)	$E_{cut}^{-3/2}$ ($eV^{-3/2}$)	nbands	$\epsilon_{max,GW\Gamma}$ (eV)	$\epsilon_{max,GW}$ (eV)
300	0.0001925	130	12.038	11.43
350	0.0001527	164	12.028	11.385
400	0.0001250	201	12.024	11.355

Highest occupied state at k-point 0: (0.000, 0.000, 0.000)

$\epsilon_{max,PBE}$: 12.45 eV

Extrapolated $\epsilon_{max,GW\Gamma@PBE}$: 12.00 eV

Extrapolated $\epsilon_{max,GW@PBE}$: 11.22 eV

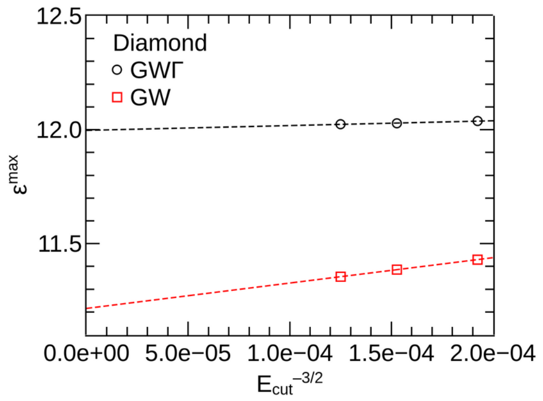

Graphite

E_{cut} (eV)	$E_{cut}^{-3/2}$ ($eV^{-3/2}$)	nbands	$\epsilon_{max,GW\Gamma}$ (eV)	$\epsilon_{max,GW}$ (eV)
300	0.0001925	423	5.898	5.259
350	0.0001527	534	5.895	5.236
400	0.0001250	652	5.901	5.221

Highest occupied state at k-point 45: (0.357, 0.357, 0.000)

$\epsilon_{max,PBE}$: 5.569 eV

Extrapolated $\epsilon_{max,GW\Gamma@PBE}$: 5.90 eV

Extrapolated $\epsilon_{max,GW@PBE}$: 5.15 eV

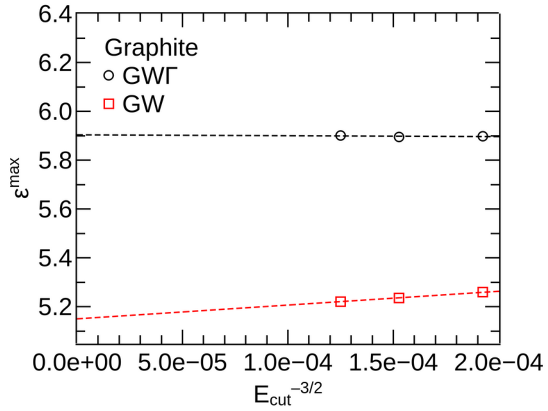

hex-BN

E_{cut} (eV)	$E_{cut}^{-3/2}$ ($eV^{-3/2}$)	nbands	$\epsilon_{max,GW\Gamma}$ (eV)	$\epsilon_{max,GW}$ (eV)
300	0.0001925	429	3.114	2.695
350	0.0001527	541	3.105	2.655
400	0.0001250	661	3.114	2.627

Highest occupied state at k-point 36: (0.357, 0.357, 0.000)

$\epsilon_{max,PBE}$: 3.95 eV

Extrapolated $\epsilon_{max,GW\Gamma@PBE}$: 3.11 eV

Extrapolated $\epsilon_{max,GW@PBE}$: 2.50 eV

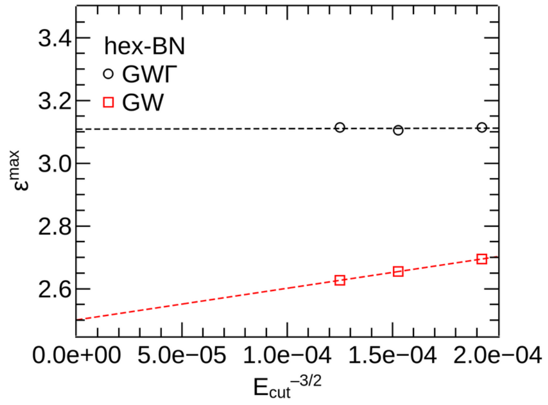

Li metal

E_{cut} (eV)	$E_{cut}^{-3/2}$ ($eV^{-3/2}$)	nbands	$\epsilon_{max,GW\Gamma}$ (eV)	$\epsilon_{max,GW}$ (eV)
300	0.0001925	497	0.288	-0.118
350	0.0001527	627	0.288	-0.119
400	0.0001250	766	0.288	-0.119

Highest occupied state at k-point 97: (0.500, 0.286, 0.214)

$\epsilon_{max,PBE}$: 0.22 eV

Extrapolated $\epsilon_{max,GW\Gamma@PBE}$: 0.29 eV

Extrapolated $\epsilon_{max,GW@PBE}$: -0.12 eV

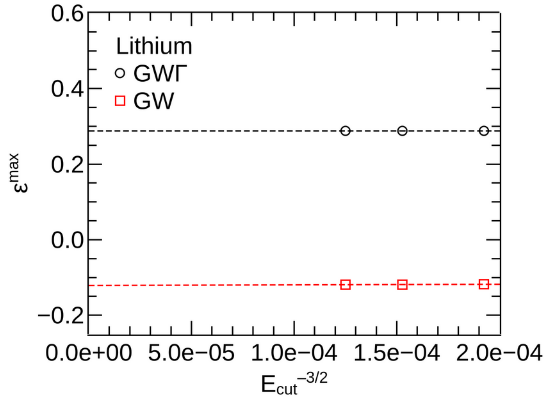

Mg metal

E_{cut} (eV)	$E_{cut}^{-3/2}$ ($eV^{-3/2}$)	nbands	$\epsilon_{max,GW\Gamma}$ (eV)	$\epsilon_{max,GW}$ (eV)
300	0.0001925	527	3.954	3.3
350	0.0001527	664	3.947	3.299
400	0.0001250	811	3.957	3.299

Highest occupied state at k-point 66: (0.357, -0.143, 0.000)

$\epsilon_{max,PBE}$: 3.75 eV

Extrapolated $\epsilon_{max,GW\Gamma@PBE}$: 3.96 eV

Extrapolated $\epsilon_{max,GW@PBE}$: 3.30 eV

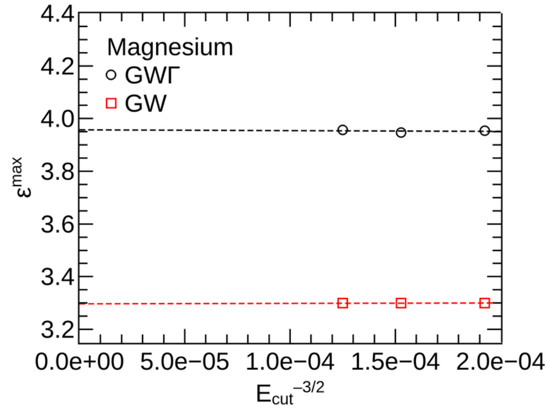

Silicon

E_{cut} (eV)	$E_{cut}^{-3/2}$ ($eV^{-3/2}$)	nbands	$\epsilon_{max,GW\Gamma}$ (eV)	$\epsilon_{max,GW}$ (eV)
300	0.0001925	473	5.301	4.652
350	0.0001527	596	5.307	4.642
400	0.0001250	728	5.303	4.634

Highest occupied state at k-point 0: (0.000, 0.000, 0.000)

$\epsilon_{max,PBE}$: 5.30 eV

Extrapolated $\epsilon_{max,GW\Gamma@PBE}$: 5.31 eV

Extrapolated $\epsilon_{max,GW@PBE}$: 4.60 eV

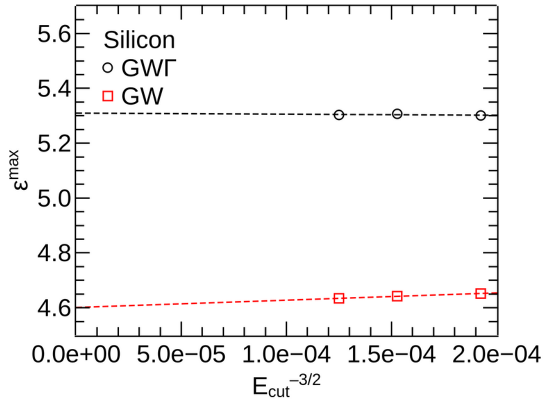

Na metal

E_{cut} (eV)	$E_{cut}^{-3/2}$ ($eV^{-3/2}$)	nbands	$\epsilon_{max,GW\Gamma}$ (eV)	$\epsilon_{max,GW}$ (eV)
300	0.0001925	869	0.326	-0.088
350	0.0001527	1095	0.326	-0.088
400	0.0001250	1338	0.327	-0.088

Highest occupied state at k-point 39: (0.417, 0.167, 0.083)

$\epsilon_{max,PBE}$: 0.22 eV

Extrapolated $\epsilon_{max,GW\Gamma@PBE}$: 0.33 eV

Extrapolated $\epsilon_{max,GW@PBE}$: -0.09 eV

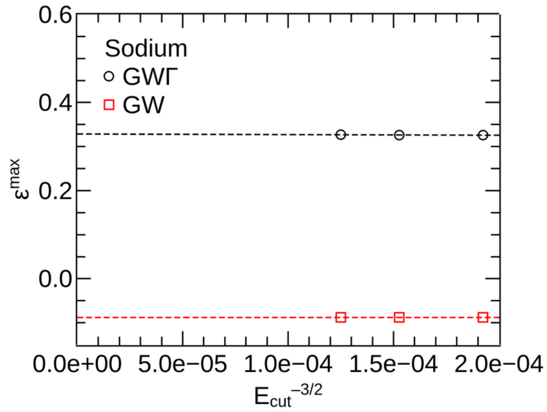

Supplement: Supplementary file 1 — ct3c00121_si_001.pdf [file ct3c00121_si_001.pdf]
